# Hepatic Energy Metabolism under the Local Control of the Thyroid Hormone System

**DOI:** 10.3390/ijms24054861

**Published:** 2023-03-02

**Authors:** Joshua Seifert, Yingfu Chen, Wenzel Schöning, Knut Mai, Frank Tacke, Joachim Spranger, Josef Köhrle, Eva Katrin Wirth

**Affiliations:** 1 Charité-Universitätsmedizin Berlin, Corporate Member of Freie Universität Berlin, Humboldt-Universität zu Berlin, and Berlin Institute of Health, Institut für Experimentelle Endokrinologie, 10115 Berlin, Germany; 2 Charité-Universitätsmedizin Berlin, Corporate Member of Freie Universität Berlin, Humboldt-Universität zu Berlin, and Berlin Institute of Health, Department of Endocrinology and Metabolism, 10115 Berlin, Germany; 3 Charité-Universitätsmedizin Berlin, Corporate Member of Freie Universität Berlin, Humboldt-Universität zu Berlin, and Berlin Institute of Health, Department of Surgery, 13353 Berlin, Germany; 4 NutriAct-Competence Cluster Nutrition Research Berlin-Potsdam, 14558 Nuthetal, Germany; 5 Charité-Universitätsmedizin Berlin, Corporate Member of Freie Universität Berlin, Humboldt-Universität zu Berlin, and Berlin Institute of Health, Department of Hepatology & Gastroenterology, Campus Virchow-Klinikum (CVK) and Campus Charité Mitte (CCM), 13353 Berlin, Germany; 6 DZHK (German Centre for Cardiovascular Research), Partner Site Berlin, 10115 Berlin, Germany

**Keywords:** T3, T4, NAFLD, energy metabolism, deiodinase, cholesterol, lipid metabolism

## Abstract

The energy homeostasis of the organism is orchestrated by a complex interplay of energy substrate shuttling, breakdown, storage, and distribution. Many of these processes are interconnected via the liver. Thyroid hormones (TH) are well known to provide signals for the regulation of energy homeostasis through direct gene regulation via their nuclear receptors acting as transcription factors. In this comprehensive review, we summarize the effects of nutritional intervention like fasting and diets on the TH system. In parallel, we detail direct effects of TH in liver metabolic pathways with regards to glucose, lipid, and cholesterol metabolism. This overview on hepatic effects of TH provides the basis for understanding the complex regulatory network and its translational potential with regards to currently discussed treatment options of non-alcoholic fatty liver disease (NAFLD) and non-alcoholic steatohepatitis (NASH) involving TH mimetics.

## 1. Introduction

Thyroid hormones (THs) are well known for their important regulatory functions during development and growth. Further early descriptions of TH actions point to the role of THs supporting homeostasis in metabolic pathways through enhancing and diminishing energy consumption from different dietary sources like carbohydrates and lipids. In addition, effects of THs in mitochondrial biogenesis and activation have been described in various models and species.

TH concentrations, mainly those of thyroxine (T4) and 3,3’,5’-triiodothyronine (T3), are assessed and accounted for mainly in the circulation where they are distributed throughout an organism. Their production and release from the thyroid gland is regulated via feedforward and feedback mechanisms within the hypothalamus-pituitary-thyroid (HPT) axis. Circulating concentrations of THs often reflect the production and release of THs mainly from the thyroid gland. However, TH actions are executed on a local tissue level within cells of the organism through direct binding to TH receptors (TRs), TRα and TRβ, acting as nuclear transcription factors (type 1 signaling) or by type 3 signaling activating signaling cascades (for example, phosphatidylinositol 3-kinase/PI3K) [1]. TR modulated gene expression in hepatocytes is mediated via TRβ, which also mediates TH signals in the HPT axis, while, for example, heart rate and brain development are mainly steered via TRα. The process of pre-receptor control determining the local TH availability within each cell is mediated by the uptake of THs into cells through transmembrane transporters and via local activation and inactivation through deiodinases (Dio). 

The eminent role of TH transmembrane transporters became obvious with the discovery of human mutations in the most specific TH transmembrane transporter, the monocarboxylate transporter (MCT) 8. These lead to the Allan-Herndon-Dudley syndrome, which is characterized by psycho-motor retardation along with high T3 and low T4 serum concentrations in patients [2,3]. MCT8 is expressed in a variety of tissues, including the brain, kidneys, and liver. In patients with mutations in MCT8 and mouse models with *Mct8* deficiency, despite the loss of Mct8 function, the liver can take up the high circulating T3 concentrations and presents a local hyperthyroid status. This indicates the presence of further TH transmembrane transporters in the liver. Transmembrane transporters that can take up THs are, for example, L-type amino acid transporters, Oatps, Mct10, and the sodium-taurocholate transporter Ntcp, which is highly expressed in the liver (since Ntcp is a major bile acid transporter). Therefore, the liver is obviously exposed to alterations in circulating TH concentrations.

Concentrations of TH in the liver are not only mediated by the uptake of circulating TH but also by local activation and inactivation through Dios. Dios are a group of selenoproteins that are responsible for the local activation and inactivation of TH metabolites via deiodination (removal of one iodide) within cells. The three different Dios are capable of reductively removing iodide from different positions within the TH molecules (for a detailed review, see [4]). While Dio2 activity is functionally relevant in the liver during development [5], the adult liver of rodents and humans displays high Dio1 activity. Dio1 activity is regulated by the presence of T3. However, Dio1 itself is involved in the activation of T4 to T3. On the other hand, it locally inactivates TH, while especially TH sulfates are substrates with high affinity [6]. Injuries to the liver, inflammation, and fasting conditions can lead to the induction of Dio3. While Dio1 is expressed in hepatocytes, up to date, it is still not known in which cells of the liver this induction takes place. While liver deiodinases regulate local availability, they also contribute to changes in circulating TH concentrations with varying contributions between different species. Additionally, the liver has a major impact on transport and stabilization of circulating TH concentrations by secreting the plasma distribution proteins albumin (binding 10–15% of T4 and 10% of T3), transthyretin (TTR, binding 10–15% of T4 and 10% of T3), and thyroxin binding globulin (TBG, binding 70% of both T4 and T3), which steady the equilibrium of free-to-total TH. The majority of THs are carried by TBG due to their much higher affinity, although TTR and albumin are much more abundant in plasma. 

While regulation of energy homeostasis in an organism is achieved by the complex interplay between different energy storing and metabolizing organs like brown and white adipose tissue, muscle, and liver, local TH action differentially affects these processes within cells of an organ. Different cell types of the same organ can even have distinct responses to local TH availability, while circulating concentrations of THs are the same throughout the organism. An example can be given through differential local T3 availability in skeletal muscle cells. Upon muscle injury, Dio3 is induced in satellite cells which promote muscle regeneration, while its inactivation leads to satellite cell apoptosis, hampering regeneration [7]. Further evaluation on a single-cell basis led to the identification of specific muscle cell subsets with differential expression and regulation of Dio2 and Dio3 upon injury and regeneration [8]. Therefore, the evaluation of local TH availability and action within an organ remains crucial. With regards to different liver cell types, the expression of Dio1, as well as TRβ, has been described many times. However, expressions of transmembrane transporters for TH, Dios, and TRs remain elusive for Kupffer and hepatic stellate cells, which also play major roles in the development of NAFLD and NASH.

In the adult organism, THs systemically regulate the energy metabolism in oxidative tissues most noticeably, so more energy-rich compounds must be supplied or mobilized from storage sites for energy production. Therefore, it is not surprising that the liver is a target organ of endocrine signaling of the TH axis, complementing the peripheral TH action. On the other hand, the liver itself is an active player in modulating TH concentrations locally and systemically by providing or removing active TH from circulation. In particular, local hepatic T3 availability orchestrates carbohydrate, lipid, cholesterol, and bile acid biogenesis at the level of gene expression, translation, and enzyme function, together with other hormones, signaling molecules, and transcription factors of nutritional regulation.

In this review, we will evaluate and summarize data from animal models and cell culture systems with regards to the impact of THs exerted on liver physiology and metabolism. To delineate between data generated in animals vs. human cell culture systems, genes and proteins of animal origin are written with only the first letter capitalized, while human genes and proteins are written in all uppercase letters. Genes are depicted in italics. Nomenclature of human and mouse genes is in accordance with HGNC and MGI. In some cases, we included data derived from human studies to either illustrate known similarities or differences between the different systems needed for the mechanistic understanding of treatment possibilities in humans. Consequences of supplying or restricting different nutrients on regulation of TH system-related genes and proteins in the liver will be discussed. In addition, we will also focus on the regulation of genes and enzymes by THs that are involved in carbohydrate, lipid, and bile acid metabolism in the liver.

## 2. Diet-Induced Alterations of the TH System

### 2.1. Fasting and Energy Restriction

Fasting influences the regulation of the hypothalamus-pituitary-thyroid (HPT) axis in various species. These physiological alterations in TH synthesis, metabolism, and action might be needed to preserve energy due to a low nutrient supply shutting down stimulatory effects of THs on energy consumption in various tissues. During fasting, a gradual decrease of serum T3 and T4 concentrations is observed, while thyroid-stimulating hormone (TSH) concentrations remain unaltered. These alterations are linked to rapidly decreased activity of hepatic Dio already observed in rat models undergoing fasting and refeeding before the individual contributions of the three Dio isoenzymes to systemic and local TH provision are distinguished [9,10] (for a recent review, see Russo et al. [11]). The data are summarized in Table 1 and Table 2.

#### 2.1.1. Systemic Alterations

According to van der Wal et al. [15], fasting resulted in a decrease of serum T3 concentrations after 12 h and serum T4 concentrations after 48 h in rats, whilst serum TSH remained constant. Lower serum concentrations of T3 and T4 (some even undetectable) were also reported upon 48-h fasting in mice [13,14] and in rats [18]. Similarly, 36-h fasting also led to decreased serum T3 and T4 concentrations in male rats [16,17] (Table 1), while three weeks of food restriction (50% of their individual baseline 24 h intake) led to a decrease only in T4 but not in T3 [17] (Table 2). Additionally, Visser et al. [21] also described that three-day fasting caused a decrease in serum T3 and T4 concentrations in both male and female rats, while TSH concentrations only decreased in male rats. Three-week food restriction (one-third of normal food intake) led to significantly lower serum T4, T3, and TSH concentrations in both male and female rats. Similar data on T4 have also been reported by Giacco et al. [20]. In a more recent study from 2020 [14], in accordance with previously reported data, 24-h fasting caused a remarkable decline in serum T4 and T3 concentrations in 12-week-old male mice. 

Moreover, fasting also affects binding of TH to plasma distribution proteins. Young et al. reported that T4 bound to Tbg and albumin increased during four-day and seven-day fasting in lean rats, while T4 bound to Ttr (designated as thyroxine-binding prealbumin, TBPA) was reduced. In obese rats, Tbg-bound T4 constantly increased up to 28 days already from day 4 upon fasting, whilst Ttr bound T4 steadily dropped and albumin bound T4 remained unaffected [22]. The fasting-induced generation of serum Tbg may account for these alterations. Moreover, serum Ttr decreased significantly during fasting, proportionally to the duration (one-, two-, and three-day fasting) in rats, accompanied by a decreased T4-bound fraction [23].

#### 2.1.2. Local Alterations in Liver

Fasting for 48 h caused a lower T3 generation rate from T4 in rat liver homogenate, indicating reduced Dio1 activity [18]. In the rat liver, T3 was lower in both 36-h fasting and three-week food restriction, whilst T4 diminished only with three-week food restriction (50% of their individual baseline 24-h intake) [17]. In mice, hepatic T4 decreased during 16- and 36-h fasting and T3 decreased upon 28- and 36-h fasting [12]. In agreement with these results, upon 24-h fasting, mice showed a decrease in hepatic T4 and T3 concentrations [14]. In male rats, hepatic TH contents were unaffected after 36-h fasting according to de Vries et al. [16]. The expression of hepatic *TR*s was not affected during fasting and food restriction, while the expression of TH responsive genes fatty acid synthase (*Fasn)* and *Spot14* was lower during fasting [17]. In addition, van der Wal et al. [15] showed that decreased serum T3 led to increased low-density lipoprotein (Ldl) cholesterol from 24 h onwards, associated with a lower liver Ldl receptor mRNA (*Ldlr*). Serum triglyceride (TG) content decreased, while serum free fatty acid (FFA) concentrations increased. Timing of fasting-induced alterations in TH availability therefore differs between species. 

The clearance of THs plays a major role in regulating the energy metabolism in a state of hunger, which is possibly responsible for the drop in TH concentrations. There are three major pathways of TH metabolism in the liver that are involved in this clearance of TH upon fasting: deiodination, sulfation, and glucuronidation. 

Deiodination contributes to the modification of TH bioactivity and availability locally via deiodinases [24,25,26]. There are two different isoforms, Dio1 and Dio3, which are capable of inactivating TH. Although Dio1 is believed to be the major source of circulating T3 in humans, the enzyme displays high affinity towards reverse T3 (rT3; 3,3′,5′-triiodo-L-thyronine) and sulfate conjugates of TH [19]. It is evident that hepatic Dio1 activity is significantly lower in both male and female rats after three-day fasting, as well as after three weeks of food restriction (one-third of normal food intake) [21]. In mice, a fasted liver showed a decrease in Dio1 activity compared with a liver in a fed state [12]. In contrast, *Dio3* mRNA expression and activity was higher during 36-h fasting and three-week food restriction (50% of their individual baseline 24-h intake), while Dio1 activity remained unchanged despite a lower mRNA expression in mice [16,17]. Thus, the fasting duration and the extent of food restriction play a role in the regulation of local TH concentrations in the liver.

To elucidate how and which deiodinases have effects on the TH concentration in both circulation and in the liver, different knockout mice of deiodinases were examined. Despite the alterations in *Dio3* expression and Dio1 activity upon fasting described by de Vries et al. [16,17], Galton et al. [12] pointed out that hepatic Dio3 activity was undetectable upon 30-h fasting in WT mice but showed minimal activity in Dio1/Dio2 double KO (D1/D2KO) mice. In accordance with above-mentioned data, serum T4 and T3 also decreased upon 30-h fasting in WT, D1KO, D2KO, and D1/D2KO mice, while TSH remained unchanged. Although D3KO showed a lower baseline of T4 and T3 than WT, 30-h fasting still caused a decline in serum T4 and T3 for D3KO as well as for WT mice. Upon fasting, rT3 is increased in D1KO and D1/D2KO mice, likely resulting from elevated Dio3 activity. Furthermore, [^125^I] labeled TH were used as Dio substrates in vivo to sensitively monitor their function during fasting. Mice were injected with either [^125^I] T4, [^125^I] T3, or [^125^I] rT3 on the same day of fasting. As a result, fasted WT mice showed a higher distribution of [^125^I] T3 in liver (and other tissues) than fed mice. In a fasted state, hepatic [^125^I] T3, [^125^I] rT3, and [^125^I] T4 were generally higher than in a fed state in WT and D1/D2KO mice, which also had increased Dio3 activity indicating alterations in uptake, efflux, and/or metabolism of THs. Taken together, fasting-induced systemic TH changes were not dependent on Dio1 or Dio2 but rather on Dio3 and sequestration of T4 and T3 in tissues but not excretion. 

Sulfation and glucuronidation are responsible for marking TH for degradation and enterohepatic recycling, and remarkable substrate preferences for the individual TH metabolites were observed for various members of the enzyme families catalyzing these conjugation reactions [19,27,28]. Sulfate conjugation may lead to the inactivation of TH by Dio1 [29] or reversible inactivation during fetal development or when TH sulfates are cleaved and liberate unconjugated TH during microbiota-dependent enterohepatic recycling [30,31,32] or cleavage by sulfatases expressed in many tissues [33,34,35]. Glucuronidation of iodothyronine facilitates its biliary and urinary excretion (reversible) [36]. Sulfotransferases (Sults) and UDP- glucuronyltransferases (Ugts) are enzymes performing sulfation and glucuronidation. Phenol sulfotransferases, which can make sulfate conjugates out of TH, belong to the Sult1 family, including Sult1a1, 1a2, 1a3, 1b1, and 1c2 [33,34,37,38]. Most UGTs involved in TH degradation are members of the Ugt1a and Ugt2b families [36,39]. It has been reported that hepatic expression of *Sultn, Sult1a1, Sult2a1,* and *Ugt1a1* increased during fasting in mice [40,41]. Reported as bilirubin UGT [21], *Ugt1a1* showed higher activity in rats upon both three-day fasting and three weeks of food restriction to one-third of normal food intake. *Sult1b1* expression upon fasting and *Sult1c1* expression with food restriction were lower in rats [17]. Fasting for 36 h induced gene expression of *Ugt1a1, Sult1a1,* and *Sult1d1* in mice, associated with an upregulation of the constitutive androstane receptor (*Car*) mRNA expression [16], and 24-h fasting induced similar effects [14]. 

*Car* is an upstream regulator of Sult and Ugt expression and a key target of nutrients, nutritional xenobiotics, and drugs interfering with hepatic metabolism both during regular nutrition and steatosis-induced alterations [42]. *Nr1l3* (*Car*) expression was upregulated during 36 h of fasting, whose serval target genes showed an upregulation during fasting to increase TH metabolism [14]. Similar induction of conjugating enzymes was achieved by *Car* agonist TCPOBOP, while these induced changes were absent in *Car*-KO mice, which showed similar TH concentrations in fed states compared with WT mice [40]. *Car^-/-^* female mice resembled WT mice upon 24-h fasting in TH concentration with lower basal and fasting-induced hepatic T3 concentrations, and they showed an attenuated induction of *Ugt1a1, Sult1a1,* and *Sult1d1*. Interestingly, *Car^-/-^* mice showed an elevated Dio3 expression and activity compared with WT mice, but the fasting-induced upregulation of *Dio3* was absent [14]. Apart from direct effects on hepatic genes related to TH metabolism and conjugation, fasting and altered leptin secretion from white adipocytes also impacted on hypothalamic neuronal circuits regulated by neuropeptide Y (Npy) and melanocortin 4 receptor (Mc4r). Such central hypothalamic inputs are also required for adaptive hepatic responses of T4 metabolizing pathways during fasting [41]. 

Apart from the local hepatic changes of TH, the uptake into and efflux of TH from the liver may play an important role in regulating systemic and local TH concentrations [43]. Evidently, 48 h of fasting led to decreased uptake of T3 and T4 into the liver, presumably due to depletion of ATP in a perfused rat liver, accompanied by diminished T3 glucuronidation [33,43]. Notably, *Mct10* expression was higher during fasting in male rats, while expression of *Mct8* remained unchanged during fasting and food restriction [17]. In mice, mRNA expression of hepatic TH transporter *Mct10* was enhanced, whilst *Mct8* expression decreased [14], similar to data obtained from rats fasting for three days [20]. In addition, 48-h fasting increased hepatic *Ntcp* mRNA expression, and 72-h fasting significantly enhanced Ntcp protein expression in the liver of rats [44]. Whether fasting-related altered expression of hepatic TH transporter transcripts is similarly reflected in changes of protein content and function, subsequently resulting in variation of the net import or export of TH, remains to be clarified.

Sirt1 is a nuclear deacetylase that is activated upon binding with ligand-bound TRß1, leading to downstream modulation of activities of various gluconeogenic transcription factors/modulators. Cordeiro et al. [13] reported that decreased TH concentrations during fasting resulted in the upregulation of Sirt1 protein and its activity via TRβ, which has beneficial effects upon 48-h fasting, for instance, on life-span extension. Glucose metabolism also plays an important role during nutrient restriction. Studies pointed out that hepatocytes regulated glucose metabolism via the hepatic cAMP/PKA/CREB pathway possibly involving Tsh receptors (Tshrs) [45,46,47]. Studies with cell-type specific knockouts of Tshr in hepatocytes and white adipocytes have been conducted [48,49]. So far, observations reported on the role of TSH and Tshr activation for hepatocyte metabolic function are mainly based on the hepatocyte-specific TshrKO mouse model of one research group [47] and need to be independently confirmed. 

### 2.2. Dietary Interventions

Diet-induced obesity has become increasingly prevalent worldwide in association with additional comorbidities such as diabetes, hyperlipidemia, and cardiovascular disease. Obesity is recognized as a major risk factor for non-alcoholic fatty liver disease (NAFLD) and non-alcoholic steatohepatitis (NASH). The imbalance between energy intake, storage, and expenditure plays a pivotal role in diet-induced obesity. TH are key modulators on energy metabolism, regulating both glucose and lipid metabolism. However, there is little data on the systemic and local regulation of TH during different diets (Table 3). 

Gonzalez-Ramos et al. [50] reported that six weeks of a high-fat diet (HFD) (10.2% hydrogenated coconut fat and 0.75% cholesterol) did not alter serum T3 and T4 concentrations in WT and *Nod1^-/-^* mice. However, three and six months of HFD with excessive iodine intake (15% lard, 10% yolk powder, and 79% standard laboratory powder chow; with 1200 μg/L iodine in the form of potassium iodate (KIO3) in the drinking water) led to increases of serum T3 and T4 concentrations and a decrease in TSH concentration [51]. 

In the LoCoTAct consortium and in our lab, we found that HFD induced Dio1 mRNA expression and activity already after four weeks, and this remained elevated for up to 18 weeks induced by HFD [52]. In accordance with these findings, induction of Dio1 mRNA expression and activity was observed in mice fed with a western diet (D12079B; Research Diets), supplemented with 15% weight/volume fructose in drinking water for eight or 16 weeks [53]. However, Han et al. [51] pointed out that only six months of HFD caused elevated hepatic Dio1 activity, while one month of HFD did not show any influence on TH concentrations or Dio1 activity. According to Gonzalez-Ramos et al. [50], although hepatic Dio1 activity was unaffected by HFD, *Nod1^-/^*^-^ mice showed significantly lower Dio1 activity independent of diet. 

Based on a liver transcriptome analysis [54], only 106 hepatic genes were differently regulated in male mice on a HFD by treatment with the thyromimetic TH metabolite 3,5-T2 (2.5µg/g bw; HFD: 60 kJ% fat; 9% soybean oil, 90% lard, four weeks), while 221 genes responded in mice fed normal chow (ND: 10 kJ% fat, 55% soybean oil, 44% lard). Among these, 56 genes were differently regulated in HFD mice. Strikingly, 12 genes (*Cyp1a2, Cyp39a1, Cyp46a1, Cyp51, Cyp2d9, Ces1(f,g) and 2a, Sult1b1, Slc13a3, Slc39a4, Gpx6*) involved in xenobiotic metabolism and detoxification were differentially expressed only in HFD mice. Elevated *Cyp39a1* expression and reduced *Cyp46a1* expression exclusively in HFD mice indicated that 3,5-T2 affected genes involved in bile acid synthesis in obese mice. Treatment with 3,5-T2 altered TH responsive gene expression such as upregulation of *Dio1* and downregulation of *Serpina7* (Tbg) for both HFD and a regular diet. 

In addition to factors that regulate the TH concentrations, expression of genes involved in lipid metabolism is altered in HFD mice. Wu et al. reported that C57BL/6 male mice fed with HFD (45% fat, 35% carbohydrate, 20% protein; 12 weeks) and db/db male mice showed upregulation of thyroid hormone-inducible hepatic protein (Thrsp) expression [55]. Thrsp, similar to the initially discovered rat S14 protein expressed in liver and adipocytes, has been used as a lipogenesis marker and endpoint of T3 action, as it rapidly responds to nutritional changes and is regulated by TH but also by steroids and other hormonal factors [56]. In db/db mice, silencing of the hepatic *Thrsp* gene led to reduced hepatic TG content and attenuated liver steatosis, while hepatic Thrsp overexpression in C57Bl/6 mice led to increased hepatic TG and cholesterol content, as well as upregulation of lipogenesis genes such as sterol regulatory binding proteins (Srebp) *Srebf1*, *Fasn* and *Acc*, associated with elevated enzyme activity. Fatty acid uptake decreased, and fatty acid oxidation increased, due to enhanced expression of peroxisome proliferator-activated receptor α (*Ppara*), acyl-CoA oxidase, and peroxisomal ketothiolase. Remarkably, treatment with TO901317 (5 mg/kg/day), a LXR agonist, led to upregulation of hepatic *Thrsp* expression, mediated by LXRα but not LXRβ [55]. According to Jornayvaz et al., TRα-0/0 mice fed with HFD (three weeks, 54.8% fat, 24% carbohydrate, 21.2% protein, energy density 4.8 Kcal/g) showed a reduction in hepatic lipid intermediates, triglyceride and DAG, as well as a decrease in expression of hepatic lipogenic genes, *Srebf1*, and its downstream targets, *Acc1* and *Fasn*. Expression of genes involved in lipid oxidation like *Fgf21*, carnitine palmitoyltransferase 1, acyl-CoA oxidase, and 70-kDa peroxisomal membrane protein mRNA levels was similar to WT [57]. 

It Is also worth mentioning that the liver is a sexually dimorphic organ [58]. Smati et al. pointed out that male mice are more susceptible to NAFLD. Male mice fed with a high-fat diet for 15 weeks (D12492, Research Diets) showed the highest lipid accumulation, and male mice fed with a western diet for 15 weeks (WD, TD.88137, Envigo) displayed the most severe inflammation/fibrosis. It is possible that the development of the disease may take much longer in females. Nevertheless, transcriptome analysis for different dietary challenges (HFD, Choline deficient-HFD (CDHFD, D05010402, Research Diets), WD and WD with glucose (18.9 g/L) and fructose (23.1 g/L) in drinking water) revealed that hepatocyte *Ppara* serves as a sexually dimorphic factor in mouse liver [59]. *Dio1*, which is altered by dietary interventions, underlies together with other selenoproteins sexual dimorphism in mouse kidneys and livers [60].

## 3. Effects of THs on Metabolic Pathways in the Liver

Circulating as well as local TH concentrations in an organism can be regulated by nutritional conditions. However, local TH also directly affect the regulation of metabolic pathways in the liver. To further elucidate mechanisms of direct canonical and non-canonical TR-mediated TH signaling in energy metabolism, a comprehensive overview of major energy pathways is presented. Since TH signaling is not always an effector in gene regulation, but a master regulator, we also introduce other selected cascades with their signal molecules and transcription factor activation in detail.

### 3.1. Carbohydrate Metabolism

Carbohydrate metabolism in the liver comprises the major energy generating pathways in the body. In this context, TH regulate basal global energy turnover and reciprocally glucose supply from stores or by recycling of energy-rich compounds in the liver.

#### 3.1.1. T3 Is the Pacemaker for Global Energy Demand

Especially during hyperthyroidism, high T3 concentrations affect glucose utilization in the whole body. One reason for the basally increased cellular energy consumption is the T3-dependent increased membrane potential, built up via Na/K-ATPases, as demonstrated in rat and hepatic rat liver cell lines [61], and by Ca2+ resequestration via Serca in mouse myocytes [62].

The glucose transporter family (Glut) supplies the raised demand for intracellular glucose in a T3-regulated manner. Weinstein et al. reported that induced hyperthyroidism in rats increased the *Glut2* transcript and protein levels in the liver [63].

#### 3.1.2. Local T3 Signal Modulation in Oxidative Tissue Affects Glucose Demand by the Liver

The liver stores glycogen as an energy reserve for extrahepatic tissues. It accumulates glucose after food intake in excess nutrient situations and releases it when blood glucose levels threaten to drop due to peripheral utilization [64]. Glucose consumption in muscle and adipose tissue is controlled locally by factors like T3 availability. Therefore, this part focuses on the component of the regulated tissue-specific T3 concentration that largely influences carbohydrate metabolism in the liver by providing energy substrates.

Intracellular deiodination of T4 to T3 mediated by Dio2 is a noteworthy element of local TH availability. While Dio2 expression and activity in the liver is only found during early development with Dio1 being the main deiodinase for local conversion of TH, in muscle and adipose tissue, this conversion is achieved by Dio2.

The rapid ubiquitination of Dio2 indicates how dynamically T3 concentration can be regulated in a tissue-specific manner and at the cellular level [65]. Salvatore et al. demonstrated by northern blotting and enzyme activity assays of human skeletal muscle biopsies that DIO2 is not only expressed but also active in skeletal muscle [66]. T3 can control glucose uptake via *Glut4* expression in muscle since the rat *Glut4* promoter shows binding affinity for TRs, as shown in the electrophoretic mobility shift assay. *Glut4* gene expression is increased in vivo after T3 administration in rats, as well as functional Glut4 transporter expression, so basal and insulin-stimulated glucose uptake are increased [67,68,69]. Thus, local Dio2 activity has a non-negligible role in insulin-stimulated glucose disposal in the muscle. *Dio2* possesses a cAMP-inducible promoter, leading to a *Dio2* expression increment after dibutyryl-cAMP treatment in rat astrocytes [70]. Moreover, bile acids can bind the G-protein coupled receptor Tgr5 that produces cAMP, which increases *Dio2* expression in mice in brown adipocytes and skeletal myocytes; thus, more available T3 increases cellular energy expenditure and protects against insulin resistance in BAT and muscle [71]. The ß_3_-adrenergic signaling due to cAMP signaling is crucial for *Dio2* expression during thermogenesis in brown adipose tissue [71].

#### 3.1.3. Gluconeogenesis Responds to Various Tissue-Specific Effects of T3 Signaling and via Direct T3 Target Genes

T3 stimulates hepatic gluconeogenesis in rats already at the level of the hypothalamic paraventricular nucleus. T3-induced sympathetic excitation influences the liver’s endogenous glucose production independently of circulating glucoregulatory hormone concentrations. This was demonstrated by subjecting rats to bilateral T3 microdialysis in the PVN, and as a result, endogenous glucose production and plasma glucose levels increased. The effect was absent when the rats underwent selective hepatic sympathectomy [72], indicating the mediating role of the sympathetic nervous system.

Local thyrotoxicosis in muscles during hyperthyroidism leads to proteolysis of muscle protein [73], with hepatic nitrogen excretion via the Cahill cycle. In this process, free ammonium is stored in alanine and transported to the liver where it undergoes deamination by alanine-aminotransferase. The resulting pyruvate is subsequently available for gluconeogenesis [74].

Gluconeogenesis is TH regulated by three rate-limiting enzymes, which provide phosphoenolpyruvate (via PCK1), control pyruvate formation (via PFK4), and the formation of D-glucose (via G6PC). T3 induces the activation of various gluconeogenic transcription factors/modulators such as FOXO1, PGC1α, ERRα, and PPAR via Sirt1 [75]. These transcription factors amplify the transcription of TH target genes, demonstrated for *PCK1*, pyruvate dehydrogenase kinase isoform 4 (*PDK4*), and *G6pc* [76,77]. It has been shown with *Sirt1* knockdown in rat livers that absent signal amplification decreases hepatic glucose production [78]. Although insulin sensitivity and disposal in skeletal muscle increases during hyperthyroidism, glucose intolerance and elevated plasma glucose concentrations have been reported in patients with Graves’ disease or non-insulin-dependent diabetes during experimental hyperthyroidism [79,80,81]. T3 signaling may trigger preferential anaerobic glycolysis, causing tissues to produce lactate rather than oxidatively degrade glucose. Pyruvate and lactate thus serve as substrates for endogenous glucose production in the liver and prevent glycogen stores from being excessively depleted by increased cellular energy expenditure [82]. However, an excessive availability of T3 overrides this protective mechanism. Battarbee et al. treated rats with 100 µg/g b.w. L-T4 and reported reduced hepatic glycogen stores, caused by T3-driven increased G6Pase activity in the hyperthyroid state [83]. The thyrotoxic enhanced enzyme activity might explain the observation by Burton et al. in 1957 when livers of rats fed 0.1% L-T4 depleted glycogen despite perfusion with high glucose concentrations in contrast to the euthyroid control group, although they had fewer glycogen stores at the start of the perfusion [84].

### 3.2. Lipid Metabolism

The thyroid state significantly affects various stages of lipid metabolism, including hepatic de novo lipogenesis. Energy consumption in the hyperthyroid organism outweighs anabolic processes over time and clears FA from the liver. Lipid metabolism in the body begins with uptake of FA from nutrition or its release from adipose tissue by lipases and circulation in the blood [85]. This chapter outlines TH regulation at the levels of FA uptake, the precursor supply from other energy metabolic pathways such as glycolysis for de novo biogenesis, and the coordination of mitochondrial FA breakdown. 

#### 3.2.1. T3 Orchestrates the Fatty Acid Uptake in a Tissue-Specific Manner

The expression of FA transporters in hepatocytes, such as FA translocase (*Fat/Cd36*), FA binding proteins (*Fabpl*), and FA transporter proteins (*Fatp*), regulate the influx of free FA into the liver. PPARs cooperate with TH-responsive genes for mobilization, degradation, and oxidation of lipids [86]. Wierzbicki et al. demonstrated the regulatory role of Ppara in the expression of *Fat/Cd36* by determining the transporter expression levels and the FA saturation status of lipids in rat liver [87].

Knock-down of FA transport protein *Fatp5* in mice after diet-induced non-alcoholic fatty liver disease (NAFLD) by HFD remarkably reduced FA uptake in the liver and reversed the NAFLD status in terms of TH content and lipid droplet formation [88]. There is certainly an influence of THs on FA uptake, while the exact mechanism needs to be further elucidated. Klieverik et al. showed that TG-derived radio-labeled FA uptake in oxidative tissue in rats increases during hyperthyroidism and decreases in BAT, whereas hypothyroidism elevates FA uptake in WAT [89]. *Fabpl* expression in the liver increases in hypothyroid rats upon T3 administration at the transcriptional and functional levels [90,91]. 

#### 3.2.2. TH Tightly Regulate Various Stages of de novo Lipogenesis via Canonical and Non-Canonical Action

In addition to uptake of FA from serum, the liver can also build FA itself for energy storage or synthesis of complex lipids. Malic enzyme (Me) forms the bridge to add energy-rich carbon compounds from glycolysis to the assembly of FA, catalyzing pyruvate to acetyl-CoA metabolism with simultaneous regeneration of one NADH equivalent. Petty et al. demonstrated in promoter studies with COS-7 cells that T3 induces *Me1* expression via a TRE [92].

**Figure 1:**
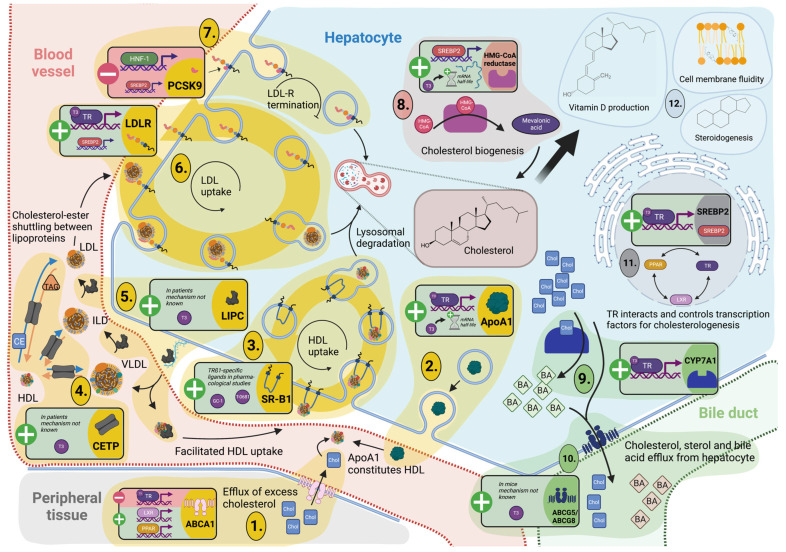
Thyroid hormone signaling controls the reverse cholesterol transport from peripheral tissues to the liver (orange) as well as the cholesterol uptake, buildup (red)**,** and turnover (green) in the liver on various rate-limiting stages. Besides the direct gene regulation, T3 signaling steers other transcription factors in the liver that mediate or amplify the T3 action (gray)**.** A profile with a schematic protein icon is given for each known regulated gene, with (+) for T3-associated upregulation and (−) for T3-associated downregulation. The mechanism, if known, is depicted on that profile. *Orchestration of transcription factors (gray)* **11**. T3 signaling not only regulates cholesterol metabolism in the liver via TR mediation but also positively influences *SREBP2* expression via type 1 regulation and the activity of other involved transcription factors, such as PPARs and LXR, via type 3 regulation. *Reverse cholesterol transport (orange)* **1**. Excess cholesterol from cells of the peripheral tissues is shuttled by cholesterol efflux regulatory protein (CERP). *ABCA1* encodes this efflux pump and is regulated negatively by T3 but positively via nuclear receptors LXRs and PPARs. Cholesterol is transported from extrahepatic tissues via plasma back to the liver by binding to high-density lipoprotein (HDL) in blood vessels. **2.** ApoAI forms the major component of HDL and is secreted by the liver. T3 upregulates *Apoa1* transcription and increases mRNA stability. **3.** HDL cholesterol can then either be taken up directly via SRB1, which has been increased in pharmacological studies with GC-1 and T-0681, or redistributed to other lipoprotein fractions. **4.** The antiport mediated by the pore-forming CETP of cholesterol esters against triglycerides between HDL, on the one hand, and VLDL, intermediate density lipoprotein (ILD), and LDL, on the other hand, describes a pathway of indirect reverse cholesterol transport. **5.** It is complemented by hepatic triglyceride lipase (encoded by LIPC) through the formation of the respective lipoprotein fractions. Both modulators are increased in serum in hyperthyroid patients, without known mechanisms. **6.** LDL cholesterol can be endocytosed at the end of this cascade by a hepatic LDL receptor. Its gene is coregulated by T3 and *SREPB2*, whereas the regulation via T3 is the determinant independent of regulation via the sterol response element. **7.** The serine protease PCSK9 circulating in serum can mediate its proteasomal termination upon binding to the LDL receptor and thus inhibit LDL uptake from serum. A negative regulation mainly via HNF1, but to a lesser extent also SREPB2, is described. *Cholesterol biogenesis (red).* The liver is the site of cholesterol biogenesis, which serves for 12 further downstream applications such as vitamin D or steroid hormone production or secretion via lipoproteins to stabilize global cellular membrane fluidity. **8.** HMG-CoA reductase provides mevalonic acid as a rate-limiting step in cholesterol buildup; it is gene regulated by SREBP2 and undergoes mRNA stabilization by T3 signaling. *Further cholesterol processing and secretion (green)* **9.** Excess cholesterol is converted to bile acid via CYP7A1, whose gene regulation is directly positively controlled by T3. **10.** The ABCG5/G8 heterodimer complex mediates secretion of cholesterol and bile acids into the bile duct. Its activity is increased by T3 in the liver without any known mechanism.

As another source for de novo lipogenesis (DNL), acetyl-CoA carboxylases (ACC) provide the conversion of acetyl-CoA from the citrate cycle to malonyl-CoA, the substrate for the assembly of long-chain amino acids. The *Acc1* isoform is present primarily in liver and adipose tissue of rats to form the substrate reservoir for the synthesis of longer-chain FA, whereas the expression of *Acc2* in oxidative tissue such as heart and skeletal muscle has a regulatory function in rats [93]. Interestingly, human RNA pools from skeletal muscle, heart, and liver, as well as skeletal muscle biopsies revealed that *ACC2* expression predominates in both lipogenic and oxidative tissues [94,95]. In chick embryo hepatocytes, the promoter of *Acc1* can bind different transcription factor complexes depending on T3 availability and thus contributes to basal expression via LXR-RXR control, whereas administration of TH could increase *Acc1* expression 7-fold [96] (Figure 1, part 11). Blennemann et al. presented, in northern blot analysis, hepatic *Acc* upregulation in hyperthyroid rats compared to hypothyroid rats [97], while data from hyperthyroid mice in comparison to euthyroid mice revealed less Acca, as well as its phosphorylated form, on the protein level [98].

Spot14 facilitates gene regulation in de novo lipogenesis. Spot14 is located in a chromosomal region associated with obesity, which phenotypically reflects the abnormal lipogenesis of *Spot14*-null mice [99]. The distance from TRE to transcription start (−2700) is enlarged compared to the usual T3-regulated genes. Studies of Campbell et al. using primary rat hepatocytes revealed that both TH and carbohydrate signaling enhanced *Spot14* expression. They proposed that the coregulating carbohydrate response element might be causal for the unexpected distance [100].

Contrary to general upregulation of the lipogenic pathway, TH negatively regulates steroyl-CoA desaturase 1 (*Scd1*), which is positively coregulated by *Srebf1*, whereas TH signaling plays the dominant role. Scd1 transforms saturated to monounsaturated fatty acids critical for complex lipid assembly of phospholipids, TG, cholesterol esters, and alkyldiacylglycerols. Experiments in HepG2 cells with co-transfection of TRß1 and RXR revealed a negative TRE in human SCD1 [101] accounting for the downregulation via THs.

Fasn generates the assembly of malonyl-CoA and acetyl-CoA to longer-chained FA such as palmitate or stearate in the presence of NADPH in the liver. Radenne et al. reported, in HepG2 cells, an increase in FASN protein expression after T3 treatment between 10 nM and 1.6 µM, which could be further increased by the additional administration of 100-nM insulin. The synergistic effect of 1.6-µM T3 and 100-nM insulin was confirmed in chicken embryo hepatocytes by a much stronger Fasn enzyme activity compared to the administration of the individual hormones. Using CAT-reporter and electrophoretic mobility shift assay, a TRE was confirmed in the goose *Fasn* 5’ UTR [102]. Furthermore, there is evidence for a non-canonical T3 action targeting the TRE by activating a PI3-kinase-ERK1/2-MAPK-dependent pathway [102]. Hönes et al. focused on the non-canonical influence of TRß on fatty acid synthesis in vivo using mouse models in which either the DNA-binding domain (*TRß^GS^*) or the domain involved in activation of PI3K were mutated (*TRß1^47F^*), as well as in TRß KO (*TRß^-/-^*) mice. After a single administration of T3 at 7 ng/g BW, comparable TG levels were reported in the WT and *TRß^GS^*, whereas the *TRß^-/-^* and *TRß^147F^* exhibited similarly increased hepatic TG. This observation was explained by increased protein levels of Fasn in the livers of animals without non-canonical TRß signaling (*TRß^-/-^* and *TRß^147F^*), whereas FASN activity in HepG2 cells was strongly reduced after treatment with T3 and PI3-kinase inhibitor LY-290042 or MEK1/2 inhibitor PD-98059 [102,103].

#### 3.2.3. TH Signaling Is the Master Regulator of Transcription Factors Controlling Hepatic Fatty Acid Metabolism

Besides direct enzymatic activity regulation to assemble FA, TH also activate lipogenic transcription factors. CHREBP, SREBF1, and LXL are noteworthy key regulators that further mediate the action of TH in the liver.

Mendoza et al. demonstrated in wild-type mice that the Chrebpa protein levels were positively influenced during hyperthyroidism while downregulated in a hypothyroid state. In *NCoR1*-KO and *NCoR1/TRß1* double knock-out mice, which provide models for altered TH action through KO of the nuclear receptor corepressor 1 (Ncor1) that directly interacts with TRs [104,105], there was no change in Chrebpa according to TH status. The positive regulation of T3-regulated genes of DNL such as *Acaca*, *Acacb*, *Fasn*, and *Me1* was absent in liver-specific *Chrebp*-KOs compared with wild types. Concentrations of two endpoint markers reflecting thyroid-state-dependent regulation (acetate for DNL and palmitate for FAO) were unaffected in the *Chrebp*-KO model, further highlighting the necessity of Chrebp for hepatic lipid metabolism. However, *Chrebp*-KO showed decreased hepatic TG concentrations during hyperthyroidism and increased concentrations in a hypothyroid situation, compared to WT. The authors proposed as an underlying mechanism that the increased hepatic TG content in the *Chrebp*-KO model in hypothyroidism is related to decreased export of VLDL from the liver, whereas in the hyperthyroid animals, the decreased FAO results from lowered availability of substrates and cofactors from DNL [106].

Downstream of the previously described TRß1-specific activation of Sirt1, the master regulator of lipogenesis, Pgc1a is activated. Sirt1 coregulates TH-signaling specific TRß1-regulated gene sets and thus mainly influences FAO, e.g., via carnitine-palmitoyltransferase-1a (*Cpt1a*) and *Pdk4* expression [75,77]. Based on a ChIP assay in rat hepatocytes, Thakran et al. proposed that via TR mediation, Sirt1 associates with the *Cpt1a* promoter to activate *Pgc1a*, which further activates *Ppara* (Figure 1, part 11). A PPAR response element (PPRE) in the first intron of *Cpt1a* is known to form the completion in the cascade from TH-mediated signal transduction to gene expression [107]. T3-induced *Ppara* signaling further leads to the expression of *Fgf21*, another transcription factor with essential effects on energy provision in the liver and adipose tissue in mice [108]. Fgf21 contributes to the enhancement of the mitochondrial oxidative function by activating the Ampk-Sirt1-Pgc1a-dependent pathway in adipocytes of the 3T3-L1 murine cell line and increasing total energy expenditure while decreasing hepatic TG content via downregulation of lipogenic gene expression in diet-induced obese mice [109,110].

#### 3.2.4. TH Regulate the Release of Fatty Acids from Intracellular Stores during Lipophagy

Autophagy is a self-digestion process that primarily recycles cellular fuel stores in lysosomes to generate amino acids, glucose, and FA [111]. Sinha et al. demonstrated that activated TR-mediated TH signaling increases autophagic flux in HepG2 cells after transfection with *TRA1*. Qualitative and quantitative protein-level analysis of autophagosome marker LC3-II indicated an increased autophagy activation. Likewise, they observed enhanced autophagy in hepatic cell lines AML-12, Hep3B, and Huh7 cells after administration of 1 µM T3 for 72 h. Furthermore, they elucidated the coupling of substrate-providing lipophagy to ß-oxidation. Phagophore membrane elongation in autophagic vesicles depends on ATG5, and siRNA-induced *ATG5* knock-down prevented a T3-driven increase in lipophagy in HepG2 and in mice to decreased ß-oxidation interpreted by reduced endpoint marker ß-hydroxybutyrate [112].

In the lysosome, lysosomal acid lipase (Lal) hydrolyzes TG and cholesterol. Coates et al. were the first to report that TH status controls Lal/cholesteryl ester hydrolase activity in rats. T3 administration of either 2 µg/g BW for four days or high single administration of 10 µg/g BW in euthyroid animals increased Lal enzyme activity compared to control animals. Accordingly, thyroidectomized animals exhibited a decreased Lal activity after four weeks [113]. Furthermore, direct TH signaling or transduction through transcription factor Foxo1 activated by deacetylation of Sirt1 affected the expression of protein determinants for autophagic processes [114], such as ULK1, Pink1, DAPK2, betatrophin, and LC3 [112,115,116,117,118]. TH-dependent modulation of the master transcription factor EB activity for lysosomal biogenesis and autophagy or the signaling cascade via PGC1A-CAMKK2-AMPK to inhibit mTOR signaling and activate autophagy by ULK1 phosphorylation expanded the picture of complex TH-induced lipophagy regulation [111,115].

#### 3.2.5. TH Regulate the Conversion of Fatty Acids into Building Blocks for Energy Production

The released FA are metabolized mainly in the mitochondrion to be supplied to the citric acid cycle for energy production or thermogenesis in BAT [119]. The peroxisome activates long-chain FA, where they are converted to membrane-permeable acyl-CoA [120]. Undoubtedly, there is an influence of TH on peroxisome activity, without an elucidated mechanism yet [85].

The transport of activated FA into the mitochondrial matrix is processed by CPT1α whereas shorter-chain FAs pass freely through the membrane [121]. Malonyl-CoA as the precursor of DNL downregulates FAO with a substrate-sensing mechanism by sterical hindrance of CPT1α [85]. Hyperthyroidism decreases intrahepatic malonyl-CoA levels. An electrophoretic mobility shift assay using hepatic rat nuclear extract and promoter analysis via luciferase readout led to the identification of a TRE and further transcription factor recognition sequences in the *CPT1A* promoter, consistent with the complex *CPT1A* regulation described above [122].

#### 3.2.6. TH Regulate Targets in Fatty Acid Catabolism either Directly or through Prolonged Mitochondrial Signaling Axis

The aforementioned PGC1α controls a mitochondrial signal axis and resembles the connection between TH signaling and mitochondrial homeostasis. In parallel to a number of directly regulated targets, the physiological response is mainly directed by transcription factors such as NRF1, NRF2, and coactivators [123].

Direct TH-regulated target genes necessary for ß-oxidation of FA in the mitochondrion include medium-chain acyl-CoA dehydrogenase (*Mcad*, identified in vivo in rats) [124], *Pdk4* (promotor analysis of rat gene) [76], and mitochondrial uncoupling protein 2 (*Ucp2*) (T3 treatment of thyroidectomized mice) [125] and hyperthyroid patients (gene expression analysis on fat tissue biopsies) [126].

There is evidence that the two TR isoforms preferentially regulate different lipid metabolic pathways. Fozzatti et al. observed in TRßPV mice that lipid turnover decreased, whereas the lipid content of the liver increased. In contrast, they described a decrease in lipogenic gene expression and changes in liver mass in TRαPV mice [127].

### 3.3. Cholesterol Metabolism and Turnover

Lipid metabolism takes place in the liver, which also synthesizes and recycles cholesterol. The sterol cholesterol is crucial for cell integrity and provides the precursor of steroid hormones, bile acids, and vitamin D (Figure 1, part 12).

Srebps represent an interface between TH signaling, on the one hand, and cholesterol/lipid homeostasis, as well as intracellular cholesterol sensing, on the other. These transcription factors are directly dependent on TH signaling and enhance or extend the effects of T3, which could be demonstrated by Shin et al. [128]. The isoform mainly involved in cholesterologenesis, SREPB2, was shown to be positively regulated by T3 in human hepatic cell lines [129].

#### 3.3.1. TH Tightly Regulate Hepatic Cholesterol Formation and Peripheral Secretion

The β-hydroxy-β-methylglutaryl-CoA (HMG-CoA) reductase marks the rate-limiting step in the formation of cholesterol in the liver (Figure 1, part 8). THs primarily regulate HMG-CoA reductase, besides estrogen, glucagon, insulin, and glucocorticoids [130,131]. In hypophysectomized rats, T3 steers the HMG-CoA reductase activity positively [131]. While a study using kidney hamster cell cultures (BHK) showed no direct effect of T3 on expression levels, T3 does stabilize HMG-CoA reductase mRNA [132]. Nevertheless, T3 controls *Srebp2* expression and thus influences HMG-CoA reductase gene regulation via their upstream regulatory elements [130].

After synthesis, loading onto lipoproteins solubilizes cholesterol so that it can be secreted from the liver to circulate in the periphery. One way to eliminate excess cholesterol from the circulation is the T3-dependent LDL endocytosis with LDLR (Figure 1, part 6). In thyroidectomized mice, oral T3 administration (10 to 50 nmol/kg/day) for seven days could recover the Ldl serum concentrations compared to sham-operated controls. With higher T3 dosing (up to 330 nmol/kg/day), overcompensation of Ldl decrease was observed [133]. The *Ldlr* promoter in the rat hepatoma cell line H4IIE contains two functional independent TREs, of which the US-TRE at −612 exhibits increased TRß1 binding affinity. Therefore, regulation via T3 is the determinant independent of the described regulation via the sterol response element [134,135] and indicates liver-specific TR isoform signaling.

The serine protease PCSK9 impairs receptor recycling and thus the uptake of LDL-bound cholesterol into the liver by facilitating lysosomal degradation. PCSK9 antibodies have already been successfully used to treat sequelae of hypercholesterolemia [136] (Figure 1, part 7). A comparative study between hyperthyroid patients versus euthyroid patients treated with liver-selective TH-analog KB2115 emphasized the effect of TH on cholesterol metabolism. Bonde et al. reported that hyperthyroidism and treatment with KB2115 reduced plasma concentrations of PCSK9, lipoprotein cholesterol, apolipoproteins B and AI, and lipoprotein(a) [137]. In line with this, two human studies showed a positive correlation between PCSK9 and TSH and a negative correlation with free T3/free T4 [138,139]. SREPBs form the direct link between TH action and PCSK9 expression, although there is no evidence for a TRE, yet. The identified regulatory sequences of PCSK9 in HepG2 cells consist of a sterol response element (SRE) and, with higher regulatory capacity, a binding site for hepatocyte nuclear factor 1 (HNF1) [140]. Worth noting is that the effect of TSH (mainly mediated via SREPB2) might have a steering capacity on PCSK9 expression in humans and HepG2 cells [141].

#### 3.3.2. TH-Mediated Processing of Circulating Cholesterol Protects against Hypercholesterolemia

Cholesterol 7a-hydroxylation is the rate-limiting step to convert LDL-bound cholesterol to bile acids. Thus, it protects against hypercholesterolemia and its sequela, such as atherosclerosis, and bile acids provide a vehicle for dietary absorption in the intestine and serve as signaling molecules [142]. The role of TH in cholesterol degradation is already evident in subclinical hypothyroid patients, who exhibit significantly elevated serum total cholesterol and decreased bile acid concentrations [143,144]. *Ldlr* knock-out mice treated with TRß-specific thyromimetics GC-1 and KB2115 demonstrated a dramatic decrease in total cholesterol, particularly LDL bound, after ten days. Lindemann et al. also reported a remarkable increase in *Cyp7a1* transcripts and serum levels of C4, a marker of bile acid synthesis and a physiological indicator of Cyp7a1 activity [145,146]. TH steer the *CYP7A1* expression in HepG2 cells with physiological TR levels via the regulatory element (N1) [147] (Figure 1, part 9).

A heterodimer structure consisting of the two semi-transporter ATP-binding cassette subfamily G, member 5 (ABCG5) and ABCG8, is responsible for the transport of formed bile acid and sterols across the canalicular membrane of hepatocytes into the gallbladder [148] (Figure 1, part 10). However, ABCG5/8 are also present in the apical membrane of enterocytes, where they mediate transintestinal cholesterol excretion (TICE) [149]. In the study of intestinal absorption of cholesterol in hypophysectomized mice, administration of TH did not normalize *Abcg5/8* expression in the small intestine but strongly upregulated it in the liver [150].

### 3.4. Reverse Cholesterol Transport

Excess cholesterol after conversion to cholesteryl ester can be transported from the periphery back to the liver by high-density lipoproteins (HDL). This process contributes to the natural cholesterol cycle and prevents manifestation of hypercholesterolemia provoked by excessive fat intake from food and cholesterol circulation in the body via VLDL and LDL. The formation of atheromatous plaques in the arteries would be promoted without reverse cholesterol transport (RCT) back to the liver and would have fatal effects on the cardio- and cerebrovascular systems.

#### 3.4.1. Cholesterol Shuttling from Peripheral Cells Is TH Dependent

RCT initially requires a cholesterol efflux pump in peripheral cells. *ABCA1* encodes the cholesterol efflux regulatory protein (CERP) and therefore resembles the shuttle for excess cholesterol. The importance of CERP in reverse cholesterol transport is reflected by the various regulators that control its expression and activity (Figure 1, part 1). Among these regulators are metabolites and signal molecules such as fatty acids, glucose, bilirubin, and adiponectin, which act via the nuclear receptors LXRs, TRs, RXRs, and PPARs [151,152,153,154,155]. Comparative promoter analysis in HEK293 cells overexpressing LXR and TR showed that both receptors bind a classical TH response element (TRE) in the *ABCA1* promoter, with LXR leading to upregulation and TR to downregulation of *ABCA1* expression [154] (Figure 1, part 11). 

#### 3.4.2. TH Control Hepatic Lipoprotein Secretion and Uptake for Reverse Cholesterol Transport and Modulation of Lipoprotein Fractions

ApoAI forms the major component of HDL secreted by the liver. Studies in rats showed that administration of TH has a positive rapid regulatory effect on *Apoa1* transcription and increases mRNA stability [156] (Figure 1, part 2). When studied in human cell lines, promoter analysis in Huh7 cells demonstrated positive regulation [157], and the increased transcripts in HepG2 cells were presumably mainly related to increased mRNA stability [158], in a similar way to the aforementioned T3 regulation on HMG-CoA reductase. In humans, however, the TH-dependent regulation of HDL is less clear than in previous animal or cell culture models of mechanistic elucidation. In patients, HDL regulation, as well as HDL particle size and the ability to bind cholesterol esters, appear to depend on the severity of TH dysregulation [159]. Trends are toward normal to high HDL in overt, normal to low in subclinical hypothyroidism, and normal to low HDL in both subclinical and overt hyperthyroidism [160,161,162]. There are additional *Apoa1* expression regulators described that act via transcription factors such as Foxa3 and epigenetic locus control with long non-coding RNA, which may explain the indistinct correlation between TH status and lipoprotein composition [163,164].

Hepatic lipase (HL) catalyzes the conversion of lipoprotein fractions from HDL via very-low-density lipoproteins (VLDL) and intermediate-low-density proteins (ILP) to low-density lipoproteins (LDL). Cholesteryl ester transport proteins (CETP) are pore forming, and they shuttle the neutral lipids cholesteryl esters and TG between lipoprotein fractions into the hydrophobic core [165] (Figure 1, part 4). HL and CETP are positively TH regulated and influence the lipoprotein composition in plasma [166]. These direct regulations via THs might be the underlying cause for increases in TG concentrations in hypothyroid individuals having low HL activity (Figure 1, part 5). Treatments with L-T4 can increase HL activity and lower TG concentrations in the circulation [167,168,169].

The uptake of the HDL-bound cholesterol ester into the liver is supported by the transporter scavenger receptor beta 1 (Srb1) (Figure 1, part 3). To date, only data on regulation are available from pharmacological studies in mice. After administration with thyromimetics GC-1 or T-0681, an increase of Srb1 at the protein level could be detected [170,171].

## 4. Future Challenges

Nutritional influences like diets and fasting impact both the systemic and the local hepatic TH system, resulting in alterations of local TH concentrations and subsequent modulation of gene expression in all pathways. With the wealth of data presented in this comprehensive overview, species- and sex-specific differences with regards to these regulations are obvious and reveal open questions. Much data has been compiled from rat models, which differ from those in mice, e.g., with regards to the extent of reduction in circulating TH concentrations upon fasting time or details of the regulation of the TH axis. Underlying molecular mechanisms fully describing the (re)distribution and enzymatic cascades involved in the clearly present reduction of circulating TH concentrations in all species remain partially elusive. Future research needs to document alterations in the liver TH system by associating different disease stages with cell-type-specific regulations of TH action, for example, for NAFLD, NASH, and diabetes models with or without local inflammation. This would provide underlying evidence for further translational insights for the possible usage and mechanism of TH mimetics that are currently in clinical trials [172,173]. While TH mimetics have been under evaluation as drugs for altering energy metabolism for many years, early substances that activated both TRα and TRß failed clinical trials due to adverse reactions. Recently, TRß-isoform-selective drugs (eliminating TRα-related adverse effects) like Resmetirom have been evaluated for the treatment of NAFLD/NASH in phase 3 clinical trials with effective reductions of hepatic and serum lipids and triglycerides. Details on thyromimetics in the context of NAFLD/NASH can be found in our recent review [172].

Nonetheless, based on the central role of local TH actions for intermediary, energy, and structural metabolism in various tissues, it is promising to therapeutically leverage the beneficial actions of THs in a tissue- and cell-type-specific manner for the treatment of metabolic disorders, including NAFLD, dyslipidemia, and obesity, as well as type 2 diabetes.

## Figures and Tables

**Table 1:** Regulation of the TH system upon fasting. N/A: not available, ↔: no change, ↓: lower than control, ↑: higher than control.

Publication	Species	Sex	Age	Fasting	Serum TSH	Serum T4	Serum T3	Liver T4	Liver T3	*Dio1* mRNA	Dio1 Activity	*Dio3* mRNA	Dio3 Activity
Galton et al., 2014, [12]	mouse	male	10–16 weeks	30 h	↔	↓	↓	N/A	N/A	N/A	N/A	N/A	N/A
Galton et al., 2014, [12]	mouse	male	10–16 weeks	36 h	N/A	N/A	N/A	↓	↓	N/A	N/A	N/A	N/A
Cordeiro et al., 2013, [13]	mouse	male	3 months	48 h	N/A	not detectable	↓	N/A	N/A	N/A	N/A	N/A	N/A
de Vries et al., 2020, [14]	mouse	male	12 weeks	48 h	N/A	↓	↓	↓	↓	N/A	N/A	↑	↔
van der Wal et al., 1998, [15]	rat	N/A	N/A	12 & 24 h	↔	↔	↓	N/A	N/A	↓	↔	N/A	N/A
de Vries et al., 2014, [16]	rat	male	N/A	36 h	N/A	↓	↓	↓	↔	↔	↔	↑	↑
de Vries et al., 2015, [17]	rat	male	8–12 weeks	36 h	N/A	↓	↓	↔	↓	↓	↔	↑	↑
Naito et al., 1981, [18]	rat	male	N/A	48 h	↓	↓	↓	lower T3 generation from T4		N/A	N/A	N/A	N/A
van der Wal et al., 1998, [15]	rat	N/A	N/A	48 h	↔	↓	↓	N/A	N/A	↓	N/A	N/A	N/A
Visser et al., 1996, [19]	rat	male	N/A	3 days	↓	↓	↓	N/A	N/A	N/A	↓	N/A	N/A
Visser et al., 1996, [19]	rat	female	N/A	3 days	↔	↓	↓	N/A	N/A	N/A	↓	N/A	N/A
Giacco et al., 2020, [20]	rat	male	3 months	66 h	N/A	↓	N/A	N/A	N/A	↓	N/A	N/A	N/A

**Table 2:** Regulation of the TH system upon food restriction. N/A: not available, ↔: no change, ↓: lower than control, ↑: higher than control.

Publication	Species	Sex	Age	Food Restriction	Duration of Restriction	Serum TSH	Serum T4	Serum T3	Liver T4	Liver T3	*Dio1* mRNA	Dio1 Activity	*Dio3* mRNA	Dio3 Activity
Visser et al., 1996, [19]	rat	male and female	N/A	one-third of normal food intake	3 weeks	↓	↓	↓	N/A	N/A	N/A	↓	N/A	N/A
de Vries et al., 2015, [17]	rat	male	8–12 weeks	50% of their individual baseline 24 h intake	21 days	N/A	↓	↓	↓	↓	↔	↔	↑	↑

**Table 3:** Regulation of the TH system through dietary interventions. N/A: not available, ↔: no change, ↓: lower than control, ↑: higher than control.

Publication	Species	Sex	Age	Genotype	Diet Composition	Duration of Dietary Intervention	Serum TSH	Serum T4	Serum T3	*Dio1* mRNA	Dio1 Activity	Other TH-Related Genes
Gonzalez-Ramos et al., 2020, [50]	mice	male	12 weeks	*Nod1^-/-^*	HFD (10.2% hydrogenated coconut fat and 0.75% cholesterol)	6 weeks	N/A	↔	↔	N/A	↓ (independent of diet)	*Glut4* ↑
Gonzalez-Ramos et al., 2020, [50]	mice	male	12 weeks	WT	HFD (10.2% hydrogenated coconut fat and 0.75% cholesterol)	6 weeks	N/A	↔	↔	N/A	↔	
Han et al., 2012, [51]	mice	female	N/A (10–13 g)	WT	HFD (15% lard, 10% yolk powder, and 79% standard laboratory powder chow; with 1200 μg/L iodine in the form of potassium iodate (KIO3) in drinking water)	6 months	↓	↑	↑	N/A	↑	
Lopez et al., 2022, [52]	mice	male	5 weeks	WT	HFD (D12492; research diets)	4–18 weeks	N/A	↓ (12 weeks)	↔	↑	↑	
Bruinstroop et al., 2021, [53]	mice	male	10 weeks	WT	WE supplemented with 15% weight/volume fructose in drinking water (D12079B; Research Diets)	8 or 16 weeks	N/A	N/A	N/A	↑	↑	
Lietzow et al., 2016, [54]	mice		20 weeks	WT	2.5 µg/g bw; HFD: 60 kJ% fat; 9% soybean oil, 90% lard, D12492, Research Diets	4 weeks	N/A	N/A	N/A	↑	N/A	*Cyp1a2, Cyp39a1, Cyp46a1, Cyp51, Cyp2d9, Ces1(f,g) and 2a, Sult1b1, Slc13a3, Slc39a4, Gpx6, Cyp39a1 ↑,Cyp46a1 ↓*

## Data Availability

Not applicable.
